# Foxo3a induces motoneuron death through the Fas pathway in cooperation with JNK

**DOI:** 10.1186/1471-2202-5-48

**Published:** 2004-11-29

**Authors:** Catherine Barthélémy, Christopher E Henderson, Brigitte Pettmann

**Affiliations:** 1 UMR 623, Developmental Biology Institute of Marseille, Centre National de la Recherche Scientifique, Institut National de la Santé et de la Recherche Médicale, Université de la Méditerranée, Assistance Publique Marseille, Campus de Luminy-Case 907, 13288 Marseille, France

## Abstract

**Background:**

Programmed cell death of motoneurons in the developing spinal cord is thought to be regulated through the availability of target-derived neurotrophic factors. When deprived of trophic support, embryonic spinal motoneurons *in vitro *over-express FasL, a ligand activating a Fas-mediated death pathway. How trophic factors regulate the expression of FasL is presently unclear, but two regulators of FasL, FOXO3a (FKHRL1) and JNK have been described to play a role in other cell types. Thus, their potential function in motoneurons was investigated in this study.

**Results:**

We show here that as a result of removal of neurotrophic factors and the consequent reduction in signalling through the PI3K/Akt pathway, Foxo3a translocates from the cytoplasm to the nucleus where it triggers cell death. Death is reduced in Fas and FasL mutant motoneurons and in the presence of JNK inhibitors indicating that a significant part of it requires activation of the Fas/FasL pathway through JNK.

**Conclusions:**

Therefore, in motoneurons as in other cell types, FOXO transcriptional regulators provide an important link between other signalling pathways and the cell death machinery.

## Background

During development of higher vertebrates, motoneurons within the spinal cord are generated in excess, and about half the cells initially generated undergo programmed cell death (PCD) during the days following target muscle contact [1]. The most frequently proposed explanation for this death is that motoneurons compete for access to limiting quantities of neurotrophic factors produced by their target tissue, and that only those which are successful survive (reviewed in [2]). Primary motoneurons purified from embryonic spinal cords and cultured in the absence of neurotrophic support mimic this process; many of them undergo programmed cell death over a period of 2–3 days [3,4]. Cell death in these conditions results from lack of activation of the survival pathways which normally inhibit the PCD machinery (reviewed in [5]). Thus, it is essential to identify the precise mechanisms by which motoneurons die, and the ways in which removal of trophic factors leads to their activation.

We have shown that a major driving force for the death of motoneurons deprived of neurotrophic factors *in vitro *is activation of the Fas/CD95 death receptor by its cognate ligand, FasL [6]. Fas and FasL are expressed by embryonic motoneurons at the stage at which naturally-occurring PCD is about to occur [6]. While levels of Fas are not affected by the presence or absence of neurotrophic factors, *FasL *expression is strongly upregulated in motoneurons cultured for 3 days without neurotrophic factors [6], as in cerebellar granule neurons [7]. Moreover, reagents such as Fas-Fc which prevent FasL from activating Fas save a majority of motoneurons from death in the absence of trophic support, presumably by blocking interactions in *cis *between FasL and Fas on individual motoneurons [6]. Understanding how expression of FasL is upregulated in motoneurons is thus an important step in linking neurotrophic signalling to cell death mechanisms.

The transcription factor Foxo3a (also known as FKHRL1) was a clear candidate. In conditions in which the PI3K/Akt survival and growth pathway is activated, Foxo3a is phosphorylated by Akt and exported to the cytoplasm where it is sequestrated by the 14-3-3 protein [8]. Overexpression of a constitutively activated form of Foxo3a (mutated at the three Akt phosphorylation sites and therefore unable to be phosphorylated) leads to PCD of many cell types in culture, including primary cerebellar granule neurons [8-14]. The *FasL *promoter contains three FOXO DNA-binding sites, and Foxo3a-induced apoptosis of cerebellar neurons is decreased when Fas/FasL interaction is blocked by the decoy fusion protein Fas-Fc [8]. Thus, in these cells, Foxo3a induces apoptosis in part by its ability to induce the expression of the *FasL *gene.

The JNK pathway has also been shown to regulate *FasL *expression in some neuronal cells, through its effects on the transcriptional activity of the AP-1 complex. Although this pathway can play different roles, in neurons it is involved in apoptosis in response to several stresses, including withdrawal of survival factors [15]. In cerebellar granule neurons, *FasL *upregulation in neurons induced to die results from JNK activation and phosphorylation of c-Jun [7]. Moreover, in cerebellar neurons derived from *gld *mice, which are defective for FasL, the killing effect induced by trophic deprivation is reduced compared to *wt *mice.

We therefore wished to study the function of Foxo3a in motoneurons and its relation to JNK signalling. We show that, in the absence of survival signalling through the Akt pathway, Foxo3a is translocated to the nucleus where it triggers motoneuron death. Using appropriate mouse mutants, we show that Fas signalling is required for roughly half of the cell death induced by Foxo3a, suggesting that *FasL *activation both directly and through JNK is a major target of its actions.

## Results

### Control of subcellular localization of Foxo3a in motoneurons

**Figure 1:**
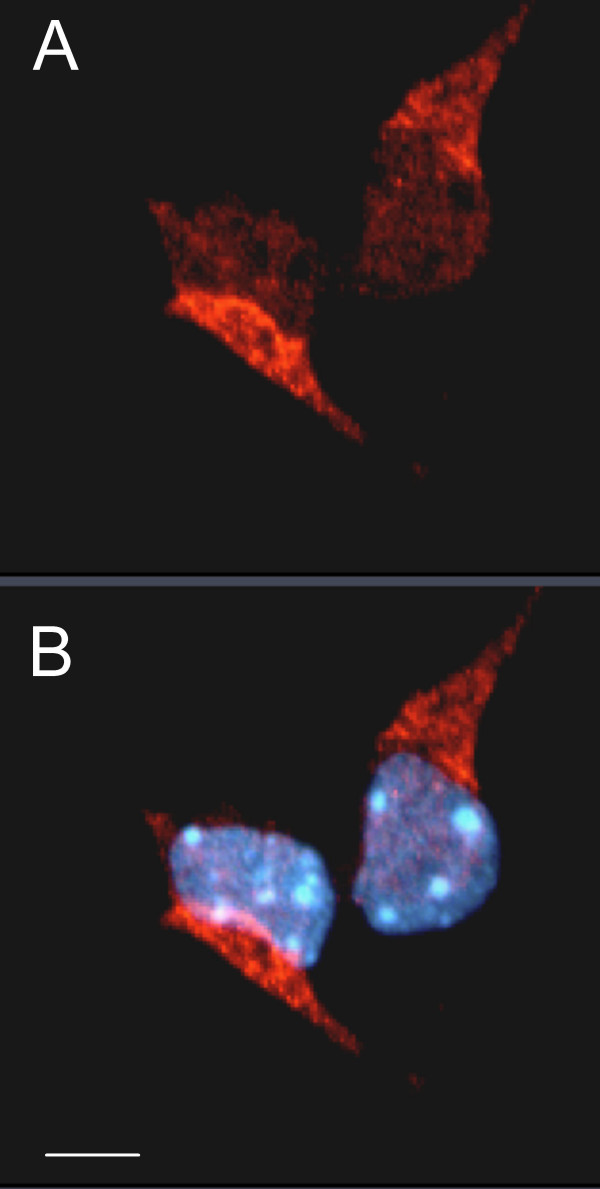
**Presence of endogenous Foxo3a in motoneurons. **Motoneurons from E12.5 mice spinal cords were cultured in the presence of a cocktail of neurotrophic factors (NTFs) and immunostained using an antibody against total Foxo3a, demonstrating a clearly cytoplasmic localization of endogenous Foxo3a in these conditions. (A) Immunolabelling of Foxo3a; (B) Immunolabelling combined with DAPI staining to visualize the nuclei. Scale bar: 5 microns

**Figure 2:**
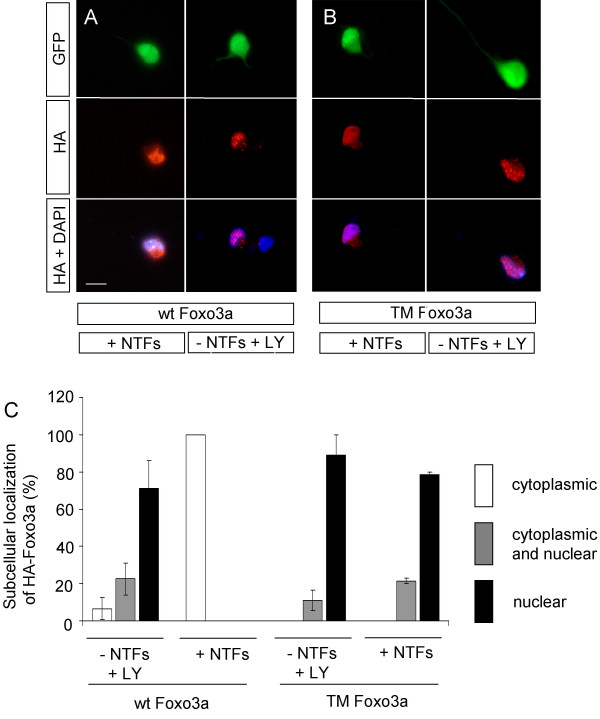
**Subcellular localization of Foxo3a reflects its state of phosphorylation by Akt. **(A, B): Motoneurons were electroporated with constructs encoding HA-tagged version of wildtype Foxo3a (wt Foxo3a; A) or the constitutively active triple-mutant form (TM Foxo3a; B), together with another vector encoding GFP. They were cultured for 24 hr either in the presence of NTFs, or in their absence. In the latter case, they were treated with the PI3K inhibitor LY 294002 (100 μM) for the last 90 min before fixation. HA-wt-Foxo3a and HA-TM-Foxo3a were detected staining for the HA tag. Characteristic labelled cells are illustrated for each condition, together with nuclear labelling using DAPI. Scale bar = 5microns. (C) Quantification of subcellular localization in A and B. Approximately 20 motoneurons were analysed in each condition in duplicate wells; results of two independent experiments are shown as mean ± range.

We first analysed the subcellular localization of Foxo3a in motoneurons using an antibody that recognizes Foxo3a independently of its phosphorylation state. Motoneurons were purified from mouse embryonic spinal cord at E12.5, at the beginning of the period of naturally-occurring cell death, and cultured in the presence of a cocktail of neurotrophic factors (NTFs; see *Methods*) to strongly activate the PI3K/Akt pathway (Perez-Garcia et al., 2004; Dolcet et al., 2001; Dolcet et al., 1999). In these conditions, Foxo3a is predominantly detected in the cytoplasm (Fig. 1). To confirm that exogenous Foxo3a adopts the same subcellular localization, we electroporated purified motoneurons with a vector encoding HA-tagged wildtype Foxo3a (HA-wt-Foxo3a) together with a vector encoding GFP to identify transduced neurons. When motoneurons were grown in the presence of NTFs, only cytoplasmic staining for HA-wt-Foxo3a was detected (Fig. 2A).

We then tested the effects of inhibiting survival signalling through the PI3K/Akt pathway by treatment with the PI3K inhibitor LY294002 either in the presence (not shown) or the absence (Fig. 2A) of neurotrophic factors. In both cases, the majority of HA-wt-Foxo3a becomes localized in the nucleus of motoneurons (Fig. 2A and not shown). This pharmacological evidence suggests that Foxo3a localization reflects its phosphorylation by Akt. To confirm this, we studied the cellular distribution of the non-phosphorylatable form of Foxo3a, triple mutant (TM-) Foxo3a, in which all three Akt phosphorylation sites are mutated (Brunet et al ref). In the absence of survival signalling, HA-TM-Foxo3a showed a very similar nuclear distribution to HA-wt-Foxo3a (Fig. 2B). However, in contrast with the wildtype form, no redistribution of HA-TM-Foxo3a to the cytoplasm was observed in the presence of the cocktail of neurotrophic factors (Fig. 2B).

Results in all conditions were quantified by counting 2 independent experiments (Fig. 2C). They clearly show that, in motoneurons as in other cell types, Akt-induced phosphorylation of Foxo3a is required to prevent its accumulation in the nucleus.

### Activated Foxo3a triggers motoneuron death

**Figure 3:**
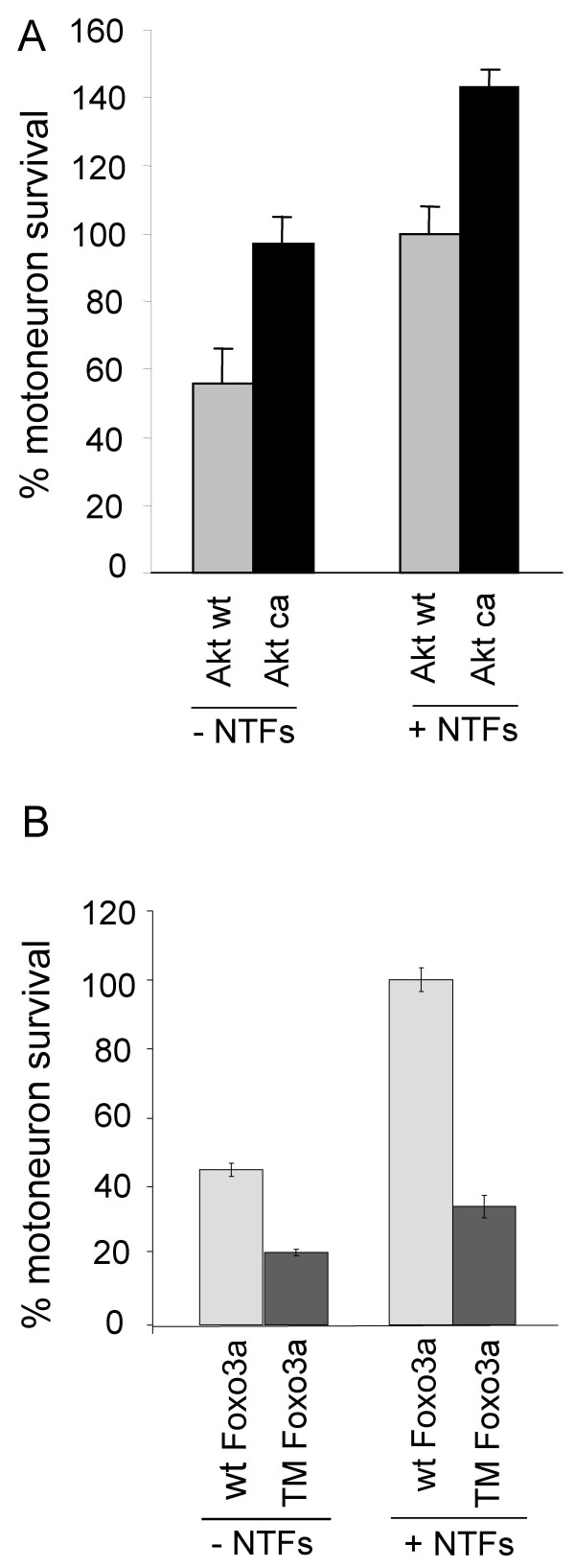
**Non-phosphorylatable triple-mutant Foxo3a triggers motoneuron death. **(A) Purified motoneurons were coelectroporated with constructs for constitutively active (ca) Akt or empty vector, together with another vector encoding GFP. The survival of transduced motoneurons was evaluated after 2 d in culture with NTFs. Akt ca further enhances the effects of NTFs on motoneuron survival. Results are mean ± SD of 2 independent experiments. (B) Using the same protocol, motoneurons were electroporated with vectors encoding either HA-wt-Foxo3a or HA-TM-Foxo3a, together with the GFP vector. They were cultured in the presence or the absence of NTFs and survival was evaluated 2 d later. Overexpression of TM-Foxo-3a leads to reduced survival in each condition. Results are mean ± SD of 12 wells from 6 independent experiments. The 100 % corresponds to the number of motoneurons electroporated with HA-wt-Foxo3a. Differences between overexpression of HA-TM-Foxo3a and HA-wt-Foxo3a were found significant using Student's t-test (p < 0.01).

To investigate the ability of Foxo3a to trigger death of cultured motoneurons, we took advantage of the electroporation technique we recently developed for high-efficacy transduction of primary neurons [16]. We first confirmed that electroporation did not intrinsically inhibit motoneuron survival, by overexpressing a constitutively active form of Akt (Akt ca) in which the PH domain is replaced by a myristyl moiety which constitutively targets Akt to the membrane. Survival of electroporated motoneurons was reproducibly increased by overexpression of Akt ca as compared to the empty vector (Fig. 3A). Indeed, survival with Akt ca was greater than with NTFs alone. This was a result of increased Akt activity, since motoneurons electroporated with wild-type Akt showed survival values no greater than those with empty vector (not shown).

We therefore used electroporation to analyse the effects of Foxo3a on motoneuron survival. Overexpression of HA-wt-Foxo3a with GFP had no significant effect as compared to GFP alone or GFP with the empty vector (GFP: 100 ± 10; GFP + HA-wt-Foxo3a: 116 ± 12). To mimic Foxo3a activation and nuclear translocation, we then overexpressed HA-TM-Foxo3a together with GFP. The triple mutant triggered significant death of motoneurons cultured with or without NTFs (Fig. 3B). In the presence of NTFs, survival was reduced by 65%, which is similar to the proportion of motoneurons that die in the absence of trophic support (Fig. 3B).

### FasL is a target of Foxo3a for motoneuron death

**Figure 4:**
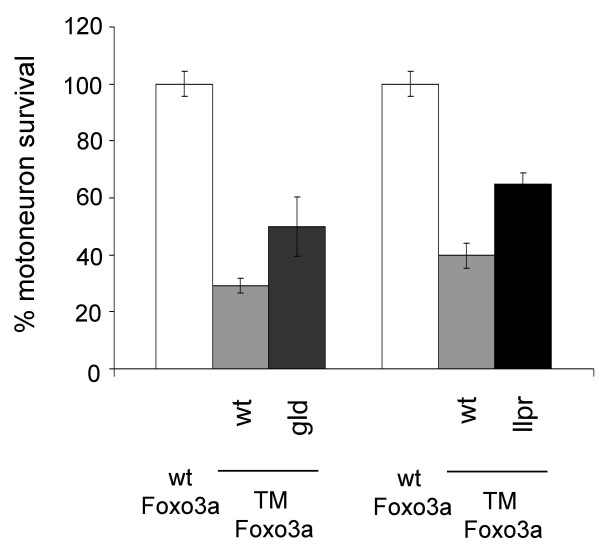
**Constitutively active Foxo3a induces motoneuron death in a Fas-dependent manner. **HA-wt-Foxo3a and HA-TM-Foxo3a were overexpressed by electroporation in control motoneurons. In parallel, HA-TM-Foxo3a was expressed in mutant motoneurons with reduced capacity for Fas signalling, *lpr *mice being deficient for Fas and *gld *mice for FasL. Cells were cultured in the presence of NTFs and grown for 2 d before counting numbers of surviving transduced motoneurons. The absence of Fas signalling reduces the ability of HA-TM Foxo3a to trigger cell death. Results are means ± SD of 4–6 wells in 2–3 independent experiments. The 100 % corresponds to the number of wt or mutant motoneurons electroporated with HA-wt-Foxo3a. Differences with the TM-Foxo3a effect in *lpr and gld *compared to control neurons were found significant using Student's t-test (p < 0.001 in *lpr *and in *gld*).

One candidate gene downstream of Foxo3a was *FasL*, known to be regulated by Foxo3a and shown by us to be able to trigger motoneuron death. Motoneurons were therefore isolated from mice bearing mutations that lead to reduced signalling through the Fas pathway: *gld *mice (point mutation in FasL) and *lpr *mice (regulatory defect in Fas). As before, electroporation of HA-TM-Foxo3a in control motoneurons led to loss of 60 to 70 % of them compared to HA-wt-Foxo3a (Fig. 4). This figure was reduced from 70 to 50 % in *gld *motoneurons, and from 60 to 35% in *lpr *mutants. Thus, induction of Fas signalling is responsible for approximately half of the cell killing induced by Foxo3a.

### Foxo3a acts partly through JNK to trigger motoneuron death

**Figure 5:**
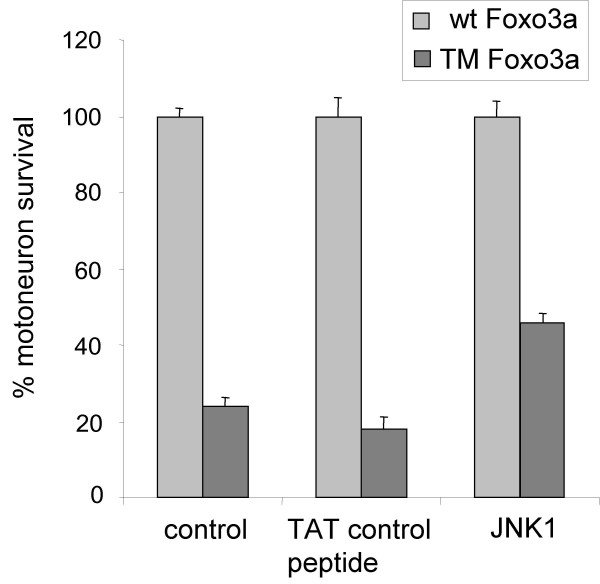
**The killing effect of TM Foxo3a involves the JNK pathway.**Motoneurons were electroporated with HA-wt-Foxo3a and cultured for 2 d with NTFs in the presence or not of a JNK inhibitor, L-JNKl1 (1 μM). Blocking the JNK pathway in motoneuron reduces the killing effect of TM-Foxo3a by about 20 %. Results are mean ± SD of 6 wells in 3 independent experiments. The 100 % corresponds to the number of motoneurons electroporated with HA-wt-Foxo3a and untreated. Differences with the TM-Foxo3a effect in L-JNKl1-treated compared with control neurons were found significant using Student's t-test (p < 0.001).

In other cell types, Foxo3a can upregulate *FasL *either by direct interaction with the *Foxo3A *promoter [8,17] or indirectly through JNK activation [18], which itself leads to upregulation of *FasL *[7]. Motoneurons were therefore electroporated with either wild-type or TM-Foxo3a, and treated immediately after plating with an inhibitor of JNK, L-JNKI1, which has been shown to inhibit the interaction between JNK and its substrates. In the presence of NTFs, JNKI1 had no effect on the survival of motoneurons electroporated with wt Foxo3a or the empty vector. In contrast, inhibition of JNK led to a reduction of 20% in the number of motoneurons triggered to die by TM-Foxo3a (Fig. 5). Since this percentage is lower than the fraction saved by reduced Fas signalling, it is likely that both direct and indirect (through JNK) mechanisms are used by Foxo3a to trigger Fas-dependent death of motoneurons.

## Discussion

Death of motoneurons as a result of insufficient trophic support was one of the first examples of developmental PCD to be discovered, but we still only partially understand the underlying mechanisms. We show here that as a result of removal of neurotrophic factors and the consequent reduction in signalling through the PI3K/Akt pathway, Foxo3a translocates from the cytoplasm to the nucleus where it triggers cell death. A significant part of this death requires activation of the Fas/FasL pathway through JNK. Thus, in motoneurons as in other cell types, FOXO transcriptional regulators provide an important link between other signalling pathways and the cell death machinery.

We overexpressed the constitutively active mutant TM-Foxo3a in the presence of neurotrophic factors to mimic the nuclear translocation of Foxo3a in their absence. We observed a 50% reduction in death induced by TM-Foxo3a when we used motoneurons that were mutant for Fas/FasL signalling. This could in theory result through several indirect mechanisms. However, the literature on direct regulation of *FasL *by Foxo3a [8] and our earlier demonstration of upregulation of *FasL *in motoneurons deprived of trophic support [6] make it likely that TM-Foxo3a is acting through FasL in these cells. This may not only reflect direct upregulation of the *FasL *gene. The partial protection obtained using a JNK inhibitor suggests that in some motoneurons, Foxo3a acts to upregulate FasL through a parallel pathway already described in other cell types, involving JNK and the AP-1 complex [7,18]. The importance of the Fas/FasL pathway in motoneuron death during development remains to be determined. However, both *in vitro *and *in vivo*, recent studies clearly demonstrate a potential role in pathological motoneuron loss. After axotomy of the facial nerve in neonates, there is a massive loss of motoneurons over the following week. The numbers of surviving motoneurons are increased 2-fold in mice deficient for the Fas/FasL pathway [19]. *In vitro*, Fas engagement leads to activation of a motoneuron-specific signalling pathway involving p38 kinase and neuronal nitric oxide synthase. Embryonic motoneurons purified from mouse models of familial amyotrophic lateral sclerosis (ALS) show greatly exacerbated death responses to activation of this pathway [16]. It will therefore be of interest to determine whether regulation of Foxo3a plays a role in determining the responsiveness of motoneurons to cell death activation in pathological situations as well.

The killing effect of Foxo3a was not totally abolished in motoneurons from *lpr *or *gld *mice. This may in part reflect the fact that these mutants are strong hypomorphs, rather than complete nulls. For example, in *lpr *mice, some Fas is still synthesized following splicing-out of the inhibitory transposon from primary transcripts [20]. However, it is equally likely that Foxo3a induces death of some motoneurons through other, Fas-independent mechanisms. Foxo3a has been shown to regulate the expression of the cyclin-dependent kinase inhibitor p27^kip1 ^[11], the glucocorticoid-induced leucine zipper protein [21], transforming growth factor-b2, [22], the DNA damage-induced protein Gadd45a [23] and the ubiquitin ligase atrogin-1 [24]. Of particular interest is its well-characterized effect on expression of Bim (Bcl-2-interacting mediator of cell death), a proapoptotic member of the Bcl-2 family that contains only the BH3 domain, which allows it to bind anti-apoptotic Bcl-2 family members and neutralize their function.

During death of sympathetic neurons induced by NGF deprivation, Foxo3a directly activates Bim expression and thereby triggers cell death [14]. Moreover, JNKs have been shown to potentiate trophic deprivation-induced apoptosis in cerebellar granule cells, through phosphorylation of Bim (Putcha et al., 2003). The following unpublished results from our laboratory make it probable that a similar mechanism occurs in purified motoneurons. Three isoforms of Bim are produced by alternative splicing in both mouse and human: Bim_S_, Bim_L _and Bim_EL _[25]. We detected all three isoforms by RT-PCR in motoneurons isolated from E12.5 ventral spinal cord of embryonic mice ([see Additional file 1], part A). Moreover, >50% of motoneurons are induced to die by overexpression of Bim_L _and 89% by overexpression of Bim_S _([see Additional file 1], part B). Therefore, expression of Bim induced by Foxo3a would be likely to trigger motoneuron death and provides the most likely explanation for the Fas-independent actions of Foxo3a in these cells.

## Conclusions

In conclusion, one of the upstream events now known to trigger death of neurons deprived of trophic support is the failure of Akt to phosphorylate Foxo3a and thereby prevent it from entering the nucleus. The fact that this mechanism is found to occur in motoneurons, a classical system for the study of neuronal cell death, opens the door to a better understanding of its role during development and in neurodegenerative pathology.

## Methods

### Animals

Normal CD1 and C57BL/6 mice were obtained from Iffacredo (L'Arbresle, France). *lpr/lpr *and *gld/gld *mice were purchased from the Jackson Laboratory (Bar Harbor, ME). *lpr/lpr *mutants present an insertion of an early transposon into the *fas *gene, resulting in *fas *transcriptional repression [20]. gld/gld mutants show a loss-of-function mutation in the fasL gene [26]. All mutants were maintained on a C57BL/6 genetic background. Controls were done in either C57BL/6 or in CD1 mice (we previously showed that their response to Fas activation was identical).

### Reagents

LY 294002 was purchased from Calbiochem and used at a final concentration of 100 μM. L-JNKl1 was purchased from Alexis Biochemicals and used at a final concentration of 1 μM. Rabbit polyclonal antibody to HA-tag was from Clontech. Antibodies against Foxo3A were as described previously [8] and were a generous gift from A. Brunet (Stanford).

### Expression constructs

We are grateful to Anne Brunet for donating vectors used as the basis for our expression constructs. The vectors encoding HA-tagged Akt and Akt ca were as described previously [27]. The vectors encoding HA-tagged wt and triple-mutant forms of Foxo3A were developed by Brunet et al[8] The cDNAs were excised from the original clones and subcloned in the pCAGGS expression vector at ClaI site, since this vector gives higher expression levels in motoneurons.

### Motoneuron purification and culture

Motoneuron cultures were prepared from E12.5 mouse spinal cords essentially as described [28], except that the magnetic column step was omitted and motoneurons in the enriched metrizamide fraction were identified by morphological criteria. Motoneurons were plated in the presence or not of a cocktail of neurotrophic factors (referred to as "NTFs": 1 ng/mL BDNF, 100 pg/mL GDNF, 10 ng/mL CNTF), added at the time of cell seeding. L-JNKl1 was added at the time of seeding, and LY294002 was added for 90 mins after 22 hours of culture.

### Electroporation of motoneurons

Cells dissociated from E12.5 mouse ventral spinal cords were centrifuged over a 6.5% (v/v) Metrizamide cushion at 2000 rpm for 15 min. Cells at the interface were collected and washed on a BSA cushion at 1500 rpm for 5 min. Cells were resuspended in electroporation buffer at a density of 50,000 cells per 100 μL. 100 μL aliquots of the suspension were transferred to 4 mm gap cuvettes (Eppendorf) and five μg of the pCAGGS-GFP vector as well as the same molar amount of the vector of interest were added. After 15 min of incubation at room temperature, cells were electroporated using three pulses of 5 ms at 200 V with intervals of 1 s. Immediately after electroporation, 1.5 ml of culture medium was added to dilute the cells which were plated in four four-wells plates [16].

### Immunocytochemistry and HA-tag localization

For anti-HA staining, motoneurons electroporated with HA-tagged wt or TM Foxo3A were seeded on polyornithine-laminin-coated 12-mm diameter glass coverslips and cultured for 24 hr at 37°C in complete Neurobasal medium supplemented or not with NTFs. Motoneurons cultured without NTFs were treated with LY294002 for the last 90 min. They were then fixed in 3.6% (v/v) formaldehyde for 30 min, washed in PBS-50 mM L-Lysine, and blocked for 1 hr in 5% donkey serum, 4% BSA, 0.1% Triton X-100 in PBS-50 mM L-Lysine. Cells were incubated with the anti-HA antibody (dilution 1:500) in blocking buffer, followed by fluorochrome-conjugated anti-rabbit secondary antibody. The cells were then observed under a fluorescence microscope. Cells with typical motoneuron morphology that were both GFP- and HA-positive were analyzed. The fraction of motoneurons expressing HA-wt-Foxo3A or HA-TM-Foxo3A exclusively in the nucleus, exclusively in the cytoplasm or in both compartments was evaluated in a total of 20 cells per well.

Staining for endogenous Foxo3A (dilution 1:100) was performed using the same method on non-electroporated motoneurons cultured for 24 hr with NTFs.

### Survival assays

Motoneurons were counted 48 hr after electroporation under a fluorescence microscope. Only green cells with healthy motoneuron morphology, i.e. with large cell bodies and long non-fragmented neurites, were taken into consideration. Two different wells for each condition were counted (between 50 and 350 green motoneurons) in all the experiments. Cross percentages of test versus control (being set at 100%) were calculated yielding 4 values, used to calculate the medium and the SD for each experiment.

## Authors' contributions

C.B. conducted most of the experiments, B.P. some. C.H and B.P. conceived the experiments and the experimental design. C.B., B.P. and C.H. wrote the paper.

## Supplementary Material

Additional File 1 **A – The 3 forms of Bim are expressed in motoneurons: **RT-PCR was performed on extracts of 80,000 motoneurones cultured for 3 days in the presence of NTFs, in a 35 mm diameter dish. Primers used for Bim were the following: 5'-GTGACAGAGAAGGTGGACAAT-3' and 5'-ATACCAGACGGAAGATAAAGC-3'. The 3 products were:BimS 284 bp, BimSL 374 bp, BimL 542 bp; **B – Overexpression of Bim_S _or Bim_L _kills a major proportion of motoneurons: **motoneurons purified from E12.5 mice embryos have been electroporated with a vector coding GFP alone or coelectroporated with a vector coding GFP and a vector coding either BimL or BimS, obtained by direct cloning of the PCR products described above first cloned into pGEMTeasy, then subcloned into pcDNA3 into EcoRI sites. Conditions of electroporation were as described in Material and Methods. The surviving electroporated motoneurons were counted after 2 days in culture in the presence of NTFs. Click here for file
