# Supplementary material for: Foxo3a induces motoneuron death through the Fas pathway in cooperation with JNK
Source: BMC Neurosci. 2004 Nov 29;5:48. doi: 10.1186/1471-2202-5-48 (PMC538283; doi:10.1186/1471-2202-5-48)
Supplement: Additional File 1 — A – The 3 forms of Bim are expressed in motoneurons: RT-PCR was performed on extracts of 80,000 motoneurones cultured for 3 days in the presence of NTFs, in a 35 mm diameter dish. Primers used for Bim were the following: 5'-GTGACAGAGAAGGTGGACAAT-3' and 5'-ATACCAGACGGAAGATAAAGC-3'. The 3 products were:BimS 284 bp, BimSL 374 bp, BimL 542 bp; B – Overexpression of BimS or BimL kills a major proportion of motoneurons: motoneurons purified from E12.5 mice embryos have been electroporated with a vector coding GFP alone or coelectroporated with a vector coding GFP and a vector coding either BimL or BimS, obtained by direct cloning of the PCR products described above first cloned into pGEMTeasy, then subcloned into pcDNA3 into EcoRI sites. Conditions of electroporation were as described in Material and Methods. The surviving electroporated motoneurons were counted after 2 days in culture in the presence of NTFs. [file 1471-2202-5-48-S1.ppt]

## Slide 1
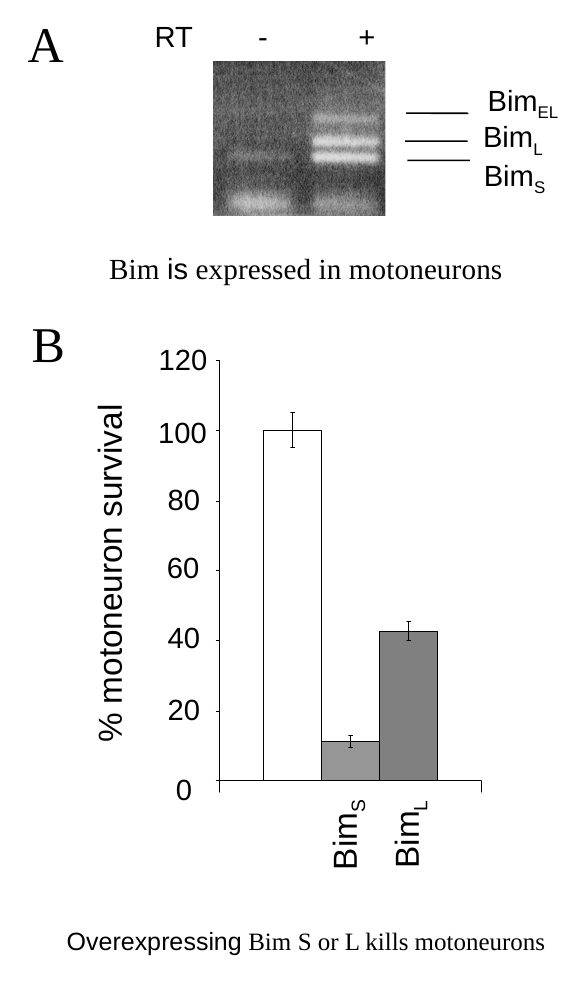

A
RT - +
BimEL
BimL
BimS
Bim is expressed in motoneurons
B
120
100
80
60
% motoneuron survival
40
20
0
BimL
BimS
Overexpressing Bim S or L kills motoneurons
